# Lung cancer in middle and southern Morocco

**DOI:** 10.3332/ecancer.2023.1518

**Published:** 2023-03-13

**Authors:** Hassan Abdelilah Tafenzi, Farah Choulli, Anass Baladi, Ismail Essaadi, Rhizlane Belbaraka

**Affiliations:** 1 Department of Medical Oncology, Mohammed VI University Hospital of Marrakech, Marrakech 40000, Morocco; 2 Biosciences and Health Laboratory, Faculty of Medicine and Pharmacy, Cadi Ayyad University, Marrakech 40000, Morocco; 3 Department of Medical Oncology, Avicenna Military Hospital of Marrakech, Marrakech 40000, Morocco

**Keywords:** lung cancer, epidemiology, overall survival

## Abstract

**Purpose:**

Determining risk factors associated with a fatal disease such as lung cancer (LC) remains an important key to understanding the factors related to its development and therefore using the correct emergent or accessible treatments. For that, we sought to highlight by describing, and analysing, the risk factors related to LC survival, reflecting the actual situation in Morocco.

**Patients and methods:**

We included 987 LC patients diagnosed from 2015 to 2021 at the Medical Oncology Department at the Mohammed VI University Hospital of Marrakech. An overview of the LC situation was described, and analysed, to determine the risk factors related to survival. The independent prognostic factors were determined using Cox Proportional Hazards Regression Analysis. To create a distinction between different risks group in the survival curve, stratification was done, respectively, within sex, age, histology type, treatments and radiation therapy.

**Results:**

We finally included 862 patients with 15 parameters among the 27 extracted, all meeting the inclusion criteria. 89.1% of the patients were male (*n* = 768) and 10.9% were female (*n* = 94), of whom 83.5% had a history of tobacco smoking (*n* = 720). The median survival of both sexes was 716 (5–2,167) days. The average age at diagnosis was 60 years. Five hundred and thirty-four patients presented with advanced stage. Patients above 66 years were the more diagnosed category with adenocarcinoma at T4N2M1c pathological category, and endocrinal comorbidity, in addition to pleurisy syndrome. Moreover, family history was found to be a bad prognostic factor. Interestingly, smoking status was not a bad contributor to survival. Age at diagnosis, histology subtype, performance status, haemoglobin, numbers of cures of the first-line chemotherapy, radiotherapy, anaemia and treatments were identified as risk factors related to survival.

**Conclusion:**

We established a descriptive and analytical overview of the current LC epidemiology situation in the oncology division of Mohammed VI University Hospital in a non-industrialised state taking into account smoking status.

## Introduction

Due to the increased prevalence of lung cancer (LC) diagnoses in both sexes over the extinct few decades, pulmonary cancer has emerged as the most lethal malignancy worldwide [1, 2].

Since 2015, LC has been the second most prevalent cancer in men in Morocco, accounting for 3,391 cases (13.3%), and it is also marked as the major cause of death, accounting for 3,157 cases (12.5%) [3]. As the occurrence varies wildly notably in developed countries, LC remains the second most common cancer in the Marrakesh-Safi region after breast cancer. Every year in the same region, approximately 2/3 of patients with LC die within 6 months of being diagnosed in the fourth stage of the disease. Because of the insufficient resources, lack of funding and the continuous use of traditional treatments, the overall survival (OS) has increased only by 5% in the last two decades in Morocco, despite the emergence of new treatments approach, and diagnoses improvements worldwide.

The increased risk of LC typically depends on the tumour, the patient and the treatment [4]. Tobacco smoking is indeed marked as a high-risk factor associated with a pulmonary cancer diagnosis; in addition, there are several other risk factors recently discovered as incidentally related to the aetiology of LC [5]. Growing evidence suggests that extra triggers may emerge from various factors in LC, including tumour genomic profiling involving molecular aberrations. Epidemiological studies in the oncology field have widely demonstrated their potential and necessity, not only in terms of trends and frequency in a given population describing the disease, but also in the analytical and clinical way to evaluate the impact of prevention strategies and design screening programmes on the overall outcome.

Thus, this paper aims to provide a variety of descriptive and causative factors to formulate the link between distribution, incidence, frequency and determinant based on several dependent variables related to patients diagnosed with LC at the Medical Oncology Department, Mohammed VI University Hospital Institution of Marrakech extracted from paper-based medical records.

## Materials and methods

This is a retrospective and descriptive study based on the Marrakesh-Safi regional registry containing a total of 987 patients admitted to the Medical Oncology Department with LC from January 2015 to December 2021 at the Cadi Ayyad university-affiliated hospital and therefore included in the study.

The extraction of patients’ related data was done manually using their medical records. The date of incidence was considered to be the date of biopsy, posterior to the date of the computed tomography scan, and therefore to the date of consultation, when the patient received confirmation of the disease. In the lost sight case, LC patients were traced using their phone numbers to ascertain their most recent survival information. All patients’ most recent medical records, including clinical examination and the recent review of computed tomography images, were used to compile follow-up data for each patient. The survival time of LC patients was calculated by subtracting the date of the last apparition or death from the biopsy date.

All information related to patients from the date of diagnosis, through clinical, pathological, biological, to treatment strategies and follow-up data were selected as inputs. It included sex (male, female), age at diagnosis, tobacco status (tobacco, tobacco + alcohol, no), biopsy date, histology type (including adenocarcinoma, neuroendocrine carcinoma (NEC), epidermoid carcinoma (EC), non-specific carcinoma (NSC), adenosquamous cell carcinoma (ASCC) and small cell carcinoma), presence or absence of any comorbidities (including cancer recurrence or relapse, cardiac, pulmonary, surgical, endocrinal, family and others), pathologic T stage, pathologic N stage, M stage, brain metastatic at diagnosis (Yes, No), medical emergency at diagnosis (including pleurisy syndrome, Pancoast-Tobias syndrome, superior vena cava syndrome and transfusion), Cooperative Oncology Group performance status, presence or absence of Epidermal Growth Factor Receptor (EGFR) mutation and Anaplastic lymphoma kinase (ALK) translocation, Programmed Death Ligand-1 (PDL-1) expression, haemoglobin, radiotherapy (Yes, No), therapeutic strategies including adjuvant, neo-adjuvant, palliative care and supportive care, chemotherapy protocol, number of the first-line chemotherapy cures, patient-reported toxicities anaemia, issues (death, disappearance or alive) and date of the last apparition. The pathologic Tumor Node Metastasis (TNM) stages were followed and characterised based on the latest edition, the 8th edition of the American Joint Committee on Cancer (AJCC) system.

Due to the lack of access to the archive of paediatric patients with LCs, since there was no mutual registry of all cancer cases in the medical institution, only patients over the age of 15 years were included in the study. In addition, patients were excluded if they were admitted to the Radiation Oncology Department with earlier stage diagnosis of LC and received only irradiation without indication of concomitant chemotherapy, and patients with missing biopsy date or survival date. Variables were not eligible for analysis if they contained more than 10% of missing values.

Continuous variables were switched into categorical variables based on quartile for age or median for the number of cures of the first-line chemotherapy and haemoglobin quantification. Univariate Cox analysis was then performed for the fitted inputs with inclusion criteria to determine the factors related to survival. A *p*-value of 0.05 or less was considered statistically significant.

The Kaplan–Meier curves were plotted to display the survival of LC patients based on sex, age at diagnosis, histology subtype, treatment-related patient and radiation therapy. The log-rank test was used to test and compare the significance hypothesis related to survival between each subgroup. All statistical analyses were done using R software (http://www.r-project.org) based on ‘survival’, ‘survminer’, ‘dplyr’, ‘devtools’, ‘tidyverse’ and ‘knitr’ packages.

## Results

### Descriptive epidemiology

The descriptive demographic and clinico-pathological characteristics of LC patients diagnosed between January 2015 and December 2021 are presented in Tables 1 and 2, which comprises 10.9% (*n* = 94) of women and 89.1% (*n* = 769) of men. For categorical variables, the description is expressed in terms of subtype numbers and percentages. Therefore, the numeric variables are expressed by minimum, maximum ranges and median as well. The highest number of patients diagnosed with LC (*n* = 256) were over 66 years old. 68% (*n* = 592) of patients had a history of Tobacco smoking and 14.8% (*n* = 128) were identified as smokers and alcoholics, however only (*n* = 131) 15.2% of patients declared their non-exposure or consumption of tobacco and/or alcohol. Four comorbidities were found to be the most related histories experienced by the same patients and included endocrinal, pulmonary, surgical and visceral related comorbidities by (*n* = 61) 7.1%, (*n* = 45) 5.2%, (*n* = 24) 2.8% and (*n* = 11) 1.3%, respectively. Adenocarcinoma 50.2% (*n* = 433) was the most common histological signature. Due to disappearance or death, 13.1% (*n* = 113) of patients did not complete the diagnosis of immunohistochemical analysis to determine the categories of NSC detected earlier. Most patients were diagnosed with T4N2M1c at the latest stage, with at least two distant metastasis and (*n* = 196) with brain metastasis at diagnosis, and were therefore declared to be admitted to palliative care. The most frequent urgencies at diagnosis were pleurisy syndrome, followed by superior cava syndrome (*n* = 39) and Pancoast syndrome (*n* = 13). 78.5% of patients did not report any urgencies at diagnosis and were admitted with the first performance status score (PS = 1). Only 39 patients were eligible for adjuvant treatment. A total of 194 patients (22.5%) underwent different types of radiation therapies depending on the issue addressed for. Regarding treatment-related toxicities, anaemia is the main toxicity reported in (*n* = 250) patients undergoing chemotherapy with a range (4, 5–10) g.dL**^−^**^1^ and 12.5 g/dL as the median. The demographic, pathologic, clinic and therapeutic characteristics of the patients enrolled in the study are listed in Tables 1 and 2. There were 279 living patients with a median follow-up time of 124 days. The median survival time was 716 days (491–1,076: 95% CI).

### Independent prognostic factors

The following variables were subjected to univariate Cox analysis: sex, age, smoking status, histology types, medical history, pathological TNM stages, brain metastasis, performance status PS, haemoglobin, number of cures of the first-line chemotherapy, anaemia, treatments and radiation therapy. As reported in Table 3, female gender (versus male *p* = 0.281) and smoking status (tobacco *p* = 0.073 and tobacco + alcohol *p* = 0.709) were not associated with prognosis. Interestingly, pathological T, N and M stages as well as brain metastasis were not highly significant in terms of survival prognosis. Meanwhile, among all histology types, adenocarcinoma subtype had the most favourable survival. Regarding comorbidities, patients with a family history of cancer (*p* = 0.0355) had a worse prognosis compared to other comorbidities. Moreover, treatment strategies were also found to be significant in terms of bad prognosis. Regarding factors associated with treatments, as shown in Table 4, patients who received radiation therapy were also found to have better survival than those who did not. Additionally, some parameters obtained during treatments, including haemoglobin rate (*p* = 0.0013), fourth grade of anaemia (*p* = 0.0346) and performance status score (*p* < 0.001) were significant. All significant results obtained by univariate Cox analysis remained prognostic factors for LC patients.

**Figure 1.**
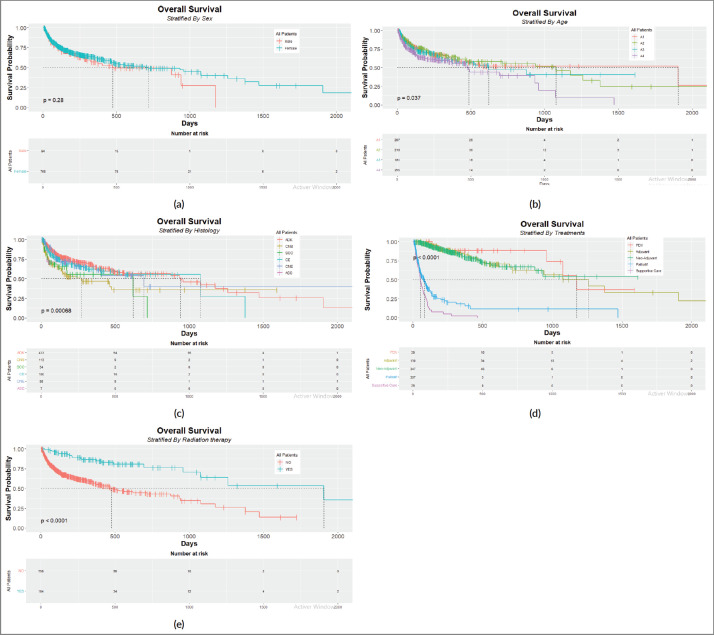
Kaplan–Meier plots with risk tables for (a): sex, (b): age, (c): histology type, (d): treatment strategy and (e): radiation therapy.

Figure 1 displays the Kaplan–Meier curves of gender, age at diagnosis, histology type, treatments and radiotherapy as prognostic factors. The log-rank test was adopted to compare each stratification subdivision.

## Discussion

Because the Kingdom of Morocco lacks a common oncology registry, and due to the absence of any other public oncology institution in middle and southern Morocco, this study focuses on a registry containing only patients admitted to the Mohammed VI University hospital with standard technical and medical care.

By analysing all demographics and clinico-pathological data, this research sought to describe the actual situation of patients admitted with LC, assessed the prognostic factors associated with death and evaluate the corresponding incidence and mortality. The cohort was obtained and built based on the medical records of patients from the Oncology Division affiliated to the Mohammed VI University Hospital as a medical institution, from 2015 to 2021. The 862 fitted patients with the inclusion criteria, the sample size of patients in this cohort and the patients’ diverse geographic distribution ensured its representativeness and generalisability for middle and southern Moroccans with LC. Through univariate Cox analysis we identified 9 factors as highly related risk factors for death from a descriptive side of the 17 variables extracted including age at diagnosis, comorbidity histories, histological cell type, performance status score, haemoglobin, number of cures from first-line chemotherapy, anaemia as toxicity, treatments related patient and radiotherapy.

As reported in many studies, LC may increasingly be described as a truly distinct mortal disease process in both sexes, women compared to men, because of a variety of endogenous and exogenous factors that may specifically contribute to a woman’s chance of acquiring it such as secondhand smoke, genetic polymorphisms and hormones replacement therapy [6–9]. Besides that, numerous research studies have shown that LC incidence strongly correlates with age, with older people having the greatest incidence rates [10, 11].

In this study, the incorporated univariate Cox analysis did not have a full literature convergence in terms of gender significance between males and females, smoking status and the OS knowing the evidence of the recent development in tumour research provides unmistakable proof that LC in women differs from LC in men. This discordance between literature and results ported by univariate Cox analysis could be explained by the late diagnosis, continuous use of traditional care and the lack of accessibility to emergent treatments including tyrosine kinase inhibitors, targeted therapy, as well as immunotherapy, therefore unceasing use of traditional chemotherapies. In addition, the scientific approach upholds the smoke acquaintance, LC incidence and mortality [12–16]. Meanwhile, patients who get cancer beyond age 66 have a bad prognosis compared with other age ranges.

Depending on the cohort studied and population characteristics, various evidence on the comorbidity effect on LC are inconsistent [17–21]. Some of them reported the impact of cardiac and pulmonary comorbidities on treatments and thus survival [19–22], while others suggest that family history of cancer, precisely LC, is associated with an increased risk of developing the disease. One study has shown that individuals with a first-degree relative with LC have a higher risk of developing the disease compared to those without a family history [23]. This risk may be even higher in individuals who have multiple family members with LC or other lung diseases.

According to the results of univariate Cox analysis, the pathological T,N,M stages as well as brain metastasis are not factors related to survival whether the tumour exceeds 7 cm, with the presence or absence of pulmonary nodules’ and having proximal or distal metastasis. We chose to consider brain metastasis as an independent metastasis factor because shreds of evidence suggest that 20%–40% of LC patients will develop brain metastasis and 10% of non-small cells LC (NSCLC) patients are diagnosed with it at first LC discovery [24–26].

According to a paper published by the AJCC about the effect of prognostic predictors on NSCLC patients, adenocarcinoma is a better significant factor among other histological types [27]. This research also found that not all histological types are related to poor prognosis, suggesting that adenocarcinoma, NEC, ASCC and EC are related to good prognosis, respectively, which confirms the AJCC paper in terms of the histological type most significant as a prognostic factor. Meanwhile, NSC and small cell are bad prognostic predictors, respectively. ASCC is known to be a rare lung histology type, with a 0.4%–4% probability of having it. According to Zhao *et al* [28] and Gawrychowski *et al* [29], when compared to other histological forms, structure-balanced adenosquamous carcinoma had often a better prognosis, while Li *et al* [30] found that ASC with foci > 5 cm is a poor prognostic predictor.

Among the results of the Cox analysis, a PS score of at least 2 was selected as a poor prognostic predictor of survival for patients receiving palliative therapy. This result was supported by recently published papers demonstrating the negative impact of PS scores on survival [31, 32]. In our case, 40 patients were declared for supportive care due to their physical situation to cope with the late-diagnosed stage of the disease. All proposed treatments are scheduled and adapted based on histology type, PS scores, pathological stages, presence of any comorbidities, presence of metastases and patient’s quality of life. It should be noted that only 39 of all LC patients benefited from surgery. Several studies have reported that not all patients are fitted with surgery [33], but those who do experienced radiotherapy and most of whom have undergone chemotherapy [34]. In addition, it has been shown by the univariate modelling approach that the number of cures of first-line chemotherapy alone can prolong the median survival and thus, is associated with a good prognosis, as well as radiation therapy. Radiotherapy has been demonstrated to significantly improve the OS in patients with T3aN2 NSCLC in retrospective analytic research using the Surveillance, Epidemiology, and End Results (SEER) database. Additionally, radiation has been shown the adding ability of 3–6 months to the median survival period of brain metastasis [35]. In fact, there is still debate concerning the prognostic significance of various therapies [35]. Since a little proportion of patients can benefit from surgery due to the absence of indication in a palliative situation, and because it is not considered a routine treatment in our institution, even if indicated, the most accessible and accurate treatments involve the combination of chemotherapy and/or radiotherapy.

Even though anaemia was not found to be fitted with inclusion criteria because it contains 50% of missing values, the main reason for including it in the analysis is that many papers discussed the relationship between the disease progression, the oncology treatments including radiotherapy, chemotherapy [36, 37], ageing and advanced stage [38], on one side, and the toxicity manifested in the patient, that in terms of statistical significance is considered to be a bad prognostic factor [39], and in terms of clinical significance, those attended to have different grades of anaemia, especially the fourth one, are more likely to never attend the OS threshold in terms of quality of life thus clinical response and therefore have a poor survival probability.

The results show that a low haemoglobin rate or anaemia, as haematological toxicity related to carboplatin-based chemotherapy, experienced by patients during treatments, particularly in the late stage, is strongly correlated with worse survival. Carboplatin and cisplatin have been recommended over two decades, as the standard regime for LC patients [40–42]. Given their haematologic toxicity and the non-haematologic toxicity often associated with their use, patients who report impaired renal function are switched to carboplatin. We did not take into account neutropenia and thrombocytopenia as haemato-toxicities factors because first, those with neutropenia take a 1–2 weeks rest in addition to antibiotic-based therapies in case of febrile neutropenia, and are supported by granulocyte growth factors. In the meantime, patients with thrombocytopenia could get a platelet-based transfusion. Secondly, there was no evidence in terms of haematological toxicities related to prognosis rather than lymphocytes/haemoglobin and lymphocytes/neutrophils ratios. In our case, not all patients’ medical records contain lymphocyte levels, thus the lymphocyte factor was not included in the study.

Oncological guidelines actualize routinely their recommendations based on the evidence that is currently available, such as the histological type of tumor, and oncogenic addiction, including tumour biomarkers and mutation signatures. The introduction of new approaches such as immune checkpoint inhibitors with their efficacy and less toxicity proven by studies and written in manuals [43, 44] makes things more complicated in developed countries including Morocco due to the financial inability of both the Moroccan state and patients to take charge of these drugs. With this lack, the department is still under the control of traditional care. If the first line of treatment fails and the new approach of immunotherapy or targeted therapy as such cannot be accessible, the old standard chemotherapy based approach is used. Patients who have multiple bone metastases receive zoledronate with the potential for ongoing radiotherapy in addition to their standard platinum-based anticancer therapy.

Comparing the results obtained in our institution with local regions, some similar Moroccan studies did not share the same finding, suggesting that the diagnosed clinical stage of the disease remains the only factor related to survival [45], while another study based on the oriental cancer database found a statistical difference between the LC incidence in men compared to women and tobacco status, comorbidities, symptoms related to the disease, clinical signs, pulmonary origin site and pleura as a metastases site were the demographic, clinic-pathological differences between the two genders related to LC incidence [46].

Okonta *et al* [47] reviewed the available Nigerian and west African sub-region data, and linked the LC situation and poorer prognosis in each country with environmental, genetic and toxic aspects, finding differences between published studies. While Soussi *et al* [48] found based on a Tunisian cohort that age at diagnosis, PS, histological type, TNM stage, with haemoglobin level, leucocyte count, calcium, lactate dehydrogenase and levels of alkaline phosphatase are the independent prognostic factors related to survival.

It is important to note that overall the prognosis for LC in Africa is poor due to factors such as late diagnosis and limited access to treatment and care [49]. Moreover, numerous studies have demonstrated that some people, particularly in North Africa, have a genetic predisposition to LC. Since North African states are a genetically diverse, complex and heterogeneous group made up of a combination of Middle Eastern Arabs, sub-Saharan Africans, Europeans and indigenous North Africans, genetic findings from the region cannot be extended to the whole of Africa [50]. The increase in the older population, particularly in the Middle East and North Africa, may be linked to improved general health care and infection control [51].

### Study limitations

Still, our research is limited by some recognised prognostic predictors (lymph nodes invasion, vascular and perineural invasions) and some important molecular parameters (epidermal growth factor receptor, anaplastic lymphoma kinase, PDL-1, ROS) not because we did not extract from patients’ records, rather that they are not routinely introduced with the immunohistochemical report until last 2021 due to the financial accessibility to the technology, and lack of financial support as well. Furthermore, efforts should be oriented towards data collection, registration and digitalisation related to patients, with the incorporation of some new molecular aberration factors related to LC.

## Conclusion

This is the first paper to publish the incidence of lung cancer data at the Marrakech Care Center. The higher incidence of men compared to women reflected in our study, as well as death, reflects the non-ability to preserve long-term survival, even in early stage, and conversely, invasive tumour cases. With the lack of innovative systemic treatments such as immunotherapy, targeted therapy and innovative imaging-guided resection, we are still under the standard archaic guidelines based on chemotherapies and therefore we could not propose a personalised treatment, tailored for each patient.

## List of abbreviations

ASCC, Adenosquamous cell carcinoma; EC, Epidermoid carcinoma; LC, Lung cancer; NEC, Neuroendocrine carcinoma; NSC, Non-specific carcinoma; NSCLC, Non-small cells lung cancer; OS, Overall survival; PDV, Perdu de Vue.

## Authors’ contributions

Conception and design: Hassan Abdelilah Tafenzi, Rhizlane Belbaraka, Ismail Essaadi

Statistical analysis: Hassan Abdelilah Tafenzi

Data interpretation: All authors

Financial support: Hassan Abdelilah Tafenzi, Rhizlane Belbaraka, Ismail Essaadi

Administrative support: Bioscience and Health Laboratory, Faculty of Medicine and Pharmacy, Cadi Ayyad University, Marrakech, Morocco & Medical Oncology Department, Mohammed VI University Hospital, Marrakech, Morocco.

Provision of study materials or patients: Hassan Abdelilah Tafenzi, Farah Choulli, Anas Baladi

Drafting: Hassan Abdelilah Tafenzi

Review, revise and approve the manuscript: All authors.

## Conflicts of interest

The authors declare no conflicts of interest.

## Funding

None.

## Ethical approval

Access to data was approved by the Ethical Review Committee of the Faculty of Medicine and Pharmacy of Marrakech. Informed consent of the patients was not required. Confidentiality was guaranteed by anonymising patient records before the analysis.

## Figures and Tables

**Table 1.** Demographic and pathologic characteristics of the cohort.

Variables	Number of patients	%
Age at diagnosis A1 = 16–54 A2 = 55–60 A3 = 61–65 A4 = Age > 66 Missing values	207 218 181 255 1	24.1 25.2 21.0 29.6 0.1
Sex Female Male	94 768	10.9 89.1
Tobacco status Tobacco Tobacco + alcohol No Missing values	592 128 130 12	68.7 14.8 15.1 1.4
Histological subtypes ADK NSC SCC EC NEC ASCC Missing values	433 113 54 180 56 7 19	50.2 13.1 6.3 20.9 6.5 0.8 2.2
Pathologic T category I II III IV Missing values	44 128 182 439 69	5.1 14.8 21.1 50.9 8
Pathologic N category N_0_ N_1_ N_2_ N_3_ Missing values	104 205 375 113 65	12.1 23.8 43.5 13.1 7.5
Pathological M category M_0_ M_1a_ M_1b_ M_1c_ Missing values	64 221 218 326 33	7.4 25.6 25.3 37.8 3.8
**Brain metastasis** No Yes	666 196	77.3 22.7

**Table 2.** Clinic and therapeutic characteristic of the cohort.

Variables	Number of patients	%
Comorbidities Cancer Cardiac Surgical Endocrinal Family Others Pulmonary No	8 8 24 66 12 13 55 676	0.9 0.9 2.8 7.7 1.4 1.5 6.4 78.4
Urgencies at diagnosis Non Others Superior vena cava syndrome Pancoast Tobias syndrome Pleurisy syndrome Transfusion Missing values	677 45 39 13 65 12 11	78.5 5.2 4.5 1.5 7.5 1.4 1.3
PS scores 1 2 3 4 Missing values	428 227 102 27 78	49.7 26.3 11.8 3.1 9
Treatments Adjuvant Neo adjuvant Palliative care PDV Supportive care	39 130 346 307 40	4.5 15.1 40.1 35.6 4.6
Radiation therapy No Yes	758 104	87.9 12.1
Protocol therapeutic No chemotherapy 5FU-Cisplatin Alimta Alimta Carboplatin Alimta Cisplatin Alimta Cisplatin + Zometa Docetaxel Docetaxel Cisplatin Etoposide Etoposide Carboplatin Etoposide Cisplatin Gemzar Gemzar - Carboplatin	353 1 2 5 30 1 2 2 2 9 44 5 29	41.0 0.1 0.2 0.6 3.5 0.1 0.2 0.2 0.2 1.0 5.1 0.6 3.4
Gemzar Cisplatin Navelbine Navelbine Carboplatin Navelbine Cisplatin Pacli Carboplatin Pacli Carboplatin + Avastin Pacli Carboplatin + Zometa Pacli Cisplatin Pacli Cisplatin + Zometa Pacli weekly Carboplatin Tarceva VAC Xeloda	39 9 52 120 131 2 1 18 1 1 1 1 1	4.5 1.0 6.0 13.9 15.2 0.2 0.1 2.1 0.1 0.1 0.1 0.1 0.1
Haematologic toxicity: Anaemia G0 G1 G2 G3 G4 Missing values	181 99 86 55 10 431	21.0 11.5 10.0 6.4 1.2 50.5
Haemoglobin Min Max Median Missing values	4.5 17.7 12.5 342	
Number of first-line chemotherapy cures Min Max Median	0 15 2	

**Table 3.** Univariate Cox proportional hazard regression analysis of demographic and pathologic characteristics.

Characteristics	HR (95% CI)	*p*-value
Sex Female Male	Reference 0.829 (0.59–1.165)	0.281
Age at diagnosis A1 = 16–54 A2 = 55–60 A3 = 61–65 A4 = Age > 66	Reference 0.943 (0.667–1.33) 1.077 (0.753–1.54) 1.436 (1.042–1.97)	0.739 0.684 0.026
Tobacco No Tobacco Tobacco + alcohol	Reference 1.37 (0.969–1.962) 1.088 (0.697–1.69)	0.073 0.709
Histology types ADK NSC SCC EC NEC ASCC	Reference 1.96 (1.41–2.73) 1.99 (1.24–3.2) 1.22 (0.894–1.682) 1.12 (0.683–1.84) 1.56 (0.385–6.321)	6.37e-05 0.00429 0.204 0.648 0.532
Pathological T stage I II III IV	Reference 1.225 (0.633–2.372) 0.827 (0.427–1.601) 1.31 (0.709–2.421)	0.546 0.573 0.388
Pathological N stage N_0_ N_1_ N_2_ N_3_	Reference 0.959 (0.623–1.479) 1.098 (0.741–1.627) 1.315 (0.828–2.091)	0.853 0.639 0.245
Pathological M stage M_0_ M_1a_ M_1b_ M_1c_	Reference 1.046 (0.633–1.726) 1.173 (0.707–1.944) 1.38 (0.856–2.24)	0.861 0.537 0.184
Brain metastasis No Yes	Reference 1.27 (0.963–1.677)	0.089

**Table 4.** Univariate Cox proportional hazard regression analysis of clinic and therapeutic characteristics.

Characteristics	HR (95% CI)	*p*-value
Comorbidities No Cancer Cardiac Chirurgical Endocrinology Family Pulmonary Others	Reference 0.39 (0.054–2.783) 1.717 (0.638–4.622) 1.142 (0.586–2.226) 0.789 (0.935–1.264) 2.392 (1.061–5.39) 1.092 (0.683–1.747) 0.717 (0.262–1.965)	0.3478 0.2840 0.6961 0.3249 0.0355 0.7122 0.5187
PS scores 1 2 3 4	Reference 2.47 (1.569–3.889) 35.04 (23.897–51.378) 69.11 (40.569–117.732)	9.35e-05 <2e-16 <2e-16
Treatment PDV Adjuvant Neo-adjuvant Palliative care Supportive care	Reference 0.064 (0.0298–0.139) 0.09 (0.0587–0.138) 0.083 (0.0596–0.115) 1.823 (1.281–2.595)	3.09e-12 <2e-16 <2e-16 0.00084
Radiotherapy No Yes	Reference 0.276 (0.172–0.442)	9.37e-08
Number of first-line chemotherapy cures	0.626 (0.542–0.724)	2.47e-10
Anaemia G0 G1 G2 G3 G4	Reference 1.091 (0.582–2.042) 1.345 (0.692–2.613) 1.281 (0.596–2.753) 3.713 (1.099–12.537)	0.7861 0.3814 0.5258 0.0346

## References

[1] Bray F, Ferlay J, Soerjomataram I (2018). Global cancer statistics 2018: GLOBOCAN estimates of incidence and mortality worldwide for 36 cancers in 185 countries. CA Cancer J Clin.

[2] Global-cancer-facts-and-figures-4th-edition.pdf. https://www.cancer.org/content/dam/cancer-org/research/cancer-facts-and-statistics/global-cancer-facts-and-figures/global-cancer-facts-and-figures-4th-edition.pdf.

[3] Haimer A, Belamalem S, Habib F (2019). Epidemiology and risk factor of lung cancer in Morocco. Biosci Biotechnol Res Asia.

[4] Rahib L, Smith BD, Aizenberg R (2014). Projecting cancer incidence and deaths to 2030: the unexpected burden of thyroid, liver, and pancreas cancers in the United States. Cancer Res.

[5] Schabath MB, Cote ML (2019). Cancer progress and priorities: lung cancer. Cancer Epidemiol Biomarkers Prev.

[6] Rivera MP (2013). Lung cancer in women: differences in epidemiology, biology, histology, and treatment outcomes. Semin Respir Crit Care Med.

[7] Barrera-Rodriguez R, Morales-Fuentes J (2012). Lung cancer in women. Lung Cancer (Auckl).

[8] Kligerman S, White C (2011). Epidemiology of lung cancer in women: risk factors, survival, and screening. AJR Am J Roentgenol.

[9] Pirie K, Peto R, Green J (2016). Lung cancer in never smokers in the UK million women study. Int J Cancer.

[10] Tamási L, Horváth K, Kiss Z (2021). Age and gender specific lung cancer incidence and mortality in Hungary: trends from 2011 through 2016. Pathol Oncol Res.

[11] Guarga L, Ameijide A, Marcos-Gragera R (2021). Trends in lung cancer incidence by age, sex and histology from 2012 to 2025 in Catalonia (Spain). Sci Rep.

[12] Youlden DR, Cramb SM, Baade PD (2008). The international epidemiology of lung cancer: geographical distribution and secular trends. J Thorac Oncol.

[13] He H, He MM, Wang H (2023). In utero and childhood/adolescence exposure to tobacco smoke, genetic risk, and lung cancer incidence and mortality in adulthood. Am J Respir Crit Care Med.

[14] Olié V, Pasquereau A, Assogba FAG (2020). Changes in tobacco-related morbidity and mortality in French women: worrying trends. Eur J Public Health.

[15] Yim SHL, Huang T, Ho JMW (2022). Rise and fall of lung cancers in relation to tobacco smoking and air pollution: a global trend analysis from 1990 to 2012. Atmos Environ.

[16] Rogers I, Memon A, Paudyal P (2022). Association between smokeless tobacco use and waterpipe smoking and the risk of lung cancer: a systematic review and meta-analysis of current epidemiological evidence. Asian Pac J Cancer Prev.

[17] Sandfeld-Paulsen B, Meldgaard P, Aggerholm-Pedersen N (2018). Comorbidity in lung cancer: a prospective cohort study of self-reported versus register-based comorbidity. J Thorac Oncol.

[18] Mellemgaard A, Lüchtenborg M, Iachina M (2015). Role of comorbidity on survival after radiotherapy and chemotherapy for nonsurgically treated lung cancer. J Thorac Oncol.

[19] Metwally EM, Rivera MP, Durham DD (2022). Lung cancer screening in individuals with and without lung-related comorbidities. JAMA Netw Open.

[20] Robinson EM, Liu BY, Sigel K (2022). Impact of comorbidities on lung cancer screening evaluation. Clin Lung Cancer.

[21] Doose M, Verhoeven D, Sanchez JI (2022). Team-based care for cancer survivors with comorbidities: a systematic review. J Healthc Qual (JHQ).

[22] Morishima T, Matsumoto Y, Koeda N (2019). Impact of comorbidities on survival in gastric, colorectal, and lung cancer patients. J Epidemiol.

[23] Guo LW, Lyu ZY, Meng QC (2022). Construction and validation of a lung cancer risk prediction model for non-smokers in China. Front Oncol.

[24] Villano JL, Durbin EB, Normandeau C (2015). Incidence of brain metastasis at initial presentation of lung cancer. Neuro Oncol.

[25] Huang Z, Hu C, Tong Y (2020). Construction of a nomogram to predict the prognosis of non-small-cell lung cancer with brain metastases. Medicine (Baltimore).

[26] Huang RSP, Harries L, Decker B (2022). Clinicopathologic and genomic landscape of non-small cell lung cancer brain metastases. Oncologist.

[27] Sculier JP, Chansky K, Crowley JJ (2008). The impact of additional prognostic factors on survival and their relationship with the anatomical extent of disease expressed by the 6th Edition of the TNM Classification of Malignant Tumors and the proposals for the 7th Edition. J Thorac Oncol.

[28] Zhao H, Yang H, Yao F (2016). Improved survival associated with a balanced structure between adenomatous and squamous components in patients with adenosquamous carcinoma of the lung. Eur J Surg Oncol (EJSO).

[29] Gawrychowski J, Bruliński K, Malinowski E (2005). Prognosis and survival after radical resection of primary adenosquamous lung carcinoma. Eur J Cardiothorac Surg.

[30] Li C, Lu H (2018). Adenosquamous carcinoma of the lung. Onco Targets Ther.

[31] Sehgal K, Gill RR, Widick P (2021). Association of performance status with survival in patients with advanced non–small cell lung cancer treated with pembrolizumab monotherapy. JAMA Netw Open.

[32] Garinet S, Wang P, Mansuet-Lupo A (2022). Updated prognostic factors in localized NSCLC. Cancers.

[33] Kamel MK, Lee B, Harrison SW (2022). Sublobar resection is comparable to lobectomy for screen-detected lung cancer. J Thorac Cardiovasc Surg.

[34] Abdel-Rahman O (2018). Outcomes of surgery as part of the management of metastatic non-small-cell lung cancer: a surveillance, epidemiology and end results database analysis. Cancer Invest.

[35] Chen D, Wang H, Song X (2018). Preoperative radiation may improve the outcomes of resectable IIIA/N2 non‐small‐cell lung cancer patients: a propensity score matching‐based analysis from surveillance, epidemiology, and end results database. Cancer Med.

[36] Zabłocka-Słowińska KA, Kosacka M, Porębska I (2017). The usefulness of routinely used malnutrition screening tools in predicting anemia in lung cancer patients. Adv Clin Exp Med.

[37] Lang E, Bissinger R, Qadri SM (2017). Suicidal death of erythrocytes in cancer and its chemotherapy: a potential target in the treatment of tumor-associated anemia. Int J Cancer.

[38] Gilreath JA, Stenehjem DD, Rodgers GM (2014). Diagnosis and treatment of cancer-related anemia. Am J Hematol.

[39] Miles LF, Cata JP, Burbury KL (2023). Anemia, thrombosis, transfusion therapy, and cancer outcomes. Perioperative Care of the Cancer Patient.

[40] Simon GR, Turrisi A (2007). Management of small cell lung cancer: ACCP evidence-based clinical practice guidelines (2nd edition). Chest.

[41] NCCN clinical practice guidelines in oncology: non small cell lung cancer – version v03.2022 national comprehensive cancer network. https://www.nccn.org/professionals/physician_gls/pdf/nscl.pdf.

[42] Rossi A, Di Maio M, Chiodini P (2012). Carboplatin- or cisplatin-based chemotherapy in first-line treatment of small-cell lung cancer: the COCIS meta-analysis of individual patient data. JCO.

[43] Escudier B, Porta C, Schmidinger M (2016). Renal cell carcinoma: ESMO clinical practice guidelines for diagnosis, treatment and follow-up. Ann Oncol.

[44] Bironzo P, Di Maio M (2018). A review of guidelines for lung cancer. J Thorac Dis.

[45] Lachgar A, Tazi MA, Afif M (2016). Lung cancer: incidence and survival in Rabat, Morocco. Rev Epidemiol Sante Publique.

[46] Belmokhtar KY, Tajir M, Boulouiz R (2019). Lung cancer in Eastern Morocco: where do we stand?. Pan Afr Med J.

[47] Okonta KE, Echieh PC, Abubakar U (2021). Management of lung cancer in Africa: underdiagnosis and poor access to treatment – a close look at Nigeria and West African Sub-region. JPATS.

[48] Soussi G, Ben Alaya N, Chaouch N (2018). Development and validation of a prognostic index for survival in non-small cell lung cancer: results from a Tunisian cohort study. Cancer Epidemiol.

[49] Barta JA, Powell CA, Wisnivesky JP (2019). Global epidemiology of lung cancer. Ann Glob Health.

[50] Arauna LR, Mendoza-Revilla J, Mas-Sandoval A (2017). Recent historical migrations have shaped the gene pool of Arabs and Berbers in North Africa. Mol Biol Evol.

[51] Gaafar R (2017). SC17.05 lung cancer in Africa: challenges and perspectives. J Thorac Oncol.

